# Effect of a novel stobadine derivative on isolated rat arteries

**DOI:** 10.2478/intox-2013-0011

**Published:** 2013-06

**Authors:** Zuzana Broskova, Ruzena Sotnikova, Jana Nedelcevova, Zsolt Bagi

**Affiliations:** 1 Institute of Experimental Pharmacology and Toxicology, Slovak Academy of Sciences, Bratislava, Slovak Republic; 2 Department of Pharmacology, University of Oxford, United Kingdom

**Keywords:** pyridoindole, SMe1EC2, aorta, arterioles

## Abstract

The antioxidant and reactive-oxygen-species-scavenging activity of stobadine has been demonstrated in previous studies. Recently, chemical modification of this leading structure led to the synthesis of other pyridoindole derivatives with significantly increased intrinsic antioxidant efficacy. Further structural modifications of stobadine provided the opportunity to increase bioavailability and attenuate unwanted side effects, such as α-adrenolytic activity. The aim of the work was to evaluate the direct effect of a novel pyridoindole, SMe1EC2, on the vascular wall ex vivo. The vasomotor effect of SMe1EC2 (1×10^–8^–1×10^–4^ mol/l) was measured on isolated and pressurized rat cerebral and coronary arterioles using video-microscopy. The effect of SMe1EC2 (1×10^–6^ and 1×10^–5^ mol/l) on high potassium-, phenylephrine- or serotonin-induced contraction or acetylcholine-induced relaxation was also determined in aortic rings. We found that SMe1EC2 (1×10^–8^–1×10^–4^ mol/l) elicited significant dilatations in both cerebral and coronary arterioles (max dilatation: 25±8% and 18±5% respectively). Yet, SMe1EC2 (1×10^–6^ and 1×10^–5^ mol/l) did not influence the tone of aortic rings nor did it affect high potassium-, phenylephrine- or serotonin -induced contractions and acetylcholine-induced relaxation. Thus SMe1EC2 was able to dilate resistance arteries but did not affect aortic contractility. It is likely that SMe1EC2 does not possess α1-adrenolytic and anti-serotoninergic activity in the vascular wall.

## Introduction

The pyridoindole antioxidant stobadine (STB) (–)-cis-2,8-dimethyl-2,3,4,4a,5,9b-hexahydro-1*H*-pyrido[4,3-b] indole has been recognized to have significant antioxidant properties and the ability to scavenge free radicals, such as hydroxyl, peroxyl, and alkoxyl radicals (Stasko *et al.*, 1990). Various *in vitro* or *in vivo* studies have shown the beneficial effect of stobadine in numerous models of oxidative-stress-involving pathologies, including myocardial infarction, stroke, neurodegenerative disorders, hypoxic-ischemic tissue injury, diabetic complications, chronic inflammation, etc. (for review see Juranek *et al.*, 2010). Series of toxicological studies including acute, subchronic and chronic toxicity tests with STB on various animals concluded that STB was safe and did not possess any severe adverse effects within the potential pharmacological range (Gajdošíková *et al.*, 1995, Dubovický *et al.*, 1999). However, a non-specific α-adrenolytic (Kvaltinova *et al.*, 1986) and H_1_-antihistaminic side effects were reported (Lukovic & Machova, 1988).

The pyridoindole antioxidant stobadine (STB) (–)-cis-2,8-dimethyl-2,3,4,4a,5,9b-hexahydro-1*H*-pyrido[4,3-b] indole has been recognized to have significant antioxidant properties and the ability to scavenge free radicals, such as hydroxyl, peroxyl, and alkoxyl radicals (Stasko *et al.*, 1990). Various *in vitro* or *in vivo* studies have shown the beneficial effect of stobadine in numerous models of oxidative-stress-involving pathologies, including myocardial infarction, stroke, neurodegenerative disorders, hypoxic-ischemic tissue injury, diabetic complications, chronic inflammation, etc. (for review see Juranek *et al.*, 2010). Series of toxicological studies including acute, subchronic and chronic toxicity tests with STB on various animals concluded that STB was safe and did not possess any severe adverse effects within the potential pharmacological range (Gajdošíková *et al.*, 1995, Dubovický *et al.*, 1999). However, a non-specific α-adrenolytic (Kvaltinova *et al.*, 1986) and H_1_-antihistaminic side effects were reported (Lukovic & Machova, 1988).

Recently, chemical modification of STB, which is considered the leading structure, led to the synthesis of pyridoindole derivatives with significantly increased intrinsic antioxidant activity and overall antioxidant efficacy compared to the parent molecule. Moreover, appropriate structural modifications of STB provided the opportunity to modulate lipophilicity and acidobasic behavior, thus optimizing bioavailability of the novel derivatives and attenuating their unwanted side effects, with the result of decreased toxicity (Stolc *et al.*, 2006). The derivative most studied so far, i.e. SMe1EC2, 2-ethoxycarbonyl-8-methoxy-2,3,4,4a,5,9b-hexahydro-1*H*-pyrido[4,3-b]indole, has already shown a potent protective effect in various animal models, such as acute head trauma (Stolc *et al.*, 2008), brain hypoxia *in vitro* (Franko *et al.*, 1999), adjuvant arthritis (Bauerova *et al.*, 2006), endothelium-mediated vasorelaxation in diabetic rats (Zurova-Nedelcevova *et al.*, 2006) or ischemia-reperfusion injury of rat hearts (Broskova & Knezl, 2011).

These findings suggest that STB derivatives might be prospectively used as medicinal antioxidants, i.e. remedies effective in conditions involving oxidative-stress-mediated injury.

The aim of this work was to determine the potential direct vasoactive effect of the novel pyridoindole derivative SMe1EC2 on isolated aortic rings as well as on isolated cerebral and coronary arterioles of the rat.

## Methods

Male Wistar rats (weighing 300–350 g; Harlan, UK or Dobrá Voda, Slovakia) were used in the experiments. The rats were housed separately, fed standard rat chow, allowed free access to drinking water, and treated according to institutional guidelines. All protocols were approved by the Institutional Animal Care and Use Committees.

### Isolated cerebral and coronary arterioles, experimental protocol

The rats were anesthetized with CO_2_, followed by subsequent cervical dislocation. Heart and brain were removed and stored in ice-cold MOPS solution. With the use of microsurgery instruments and an operating microscope, a branch of the septal coronary artery or superficial branch of the middle cerebral artery (approximately 1 mm in length) was isolated and transferred into an organ chamber containing two glass micropipettes and filled with cold MOPS solution (0–4 °C). The arteries were then cannulated on both ends on micropipettes, secured with sutures, and both micropipettes were connected with silicone tubing to an adjustable hydrostatic reservoir. The vessel chamber (15 ml) was continuously supplied with heated Krebs solution composed of (in mmol/l) NaCl 120, KCl 5.9, NaH_2_PO_4_ 1.2, MgCl_2_ 1.2, NaHCO_3_ 15.4, CaCl_2_ 2.5 (coronary)/1.25 (cerebral artery), and glucose 5.5, bubbled with 21% O_2_ and 5% CO_2_ to reach the physiological pH. The temperature was set by a temperature controller at 37 °C and the vessel was allowed to develop spontaneous tone in response to an intraluminal pressure of 60 mmHg under no flow conditions (equilibration period of 1 h). The internal arteriolar diameter (at the midpoint of the arteriolar segment) was measured by videomicroscopy (internal active diameter: coronary artery 175±8 µm, cerebral artery 128±10 µm).

After 1-h incubation period, acetylcholine (Ach) was applied abluminally in increasing concentrations (from 1×10^–8^ to maximum 1×10^–5^ mol/l) to test the viability of the vessel. Afterwards, SMe1EC2.2HCl was applied in increasing concentrations (1×10^–8^ to 1×10^–4^ mol/l). Arteriolar responses to agonists were expressed as percentage of the maximal dilation of the vessel.

### Isolated aorta, experimental protocol

The rat thoracic aorta was excised and transferred into oxygenated physiological salt solution (PSS). The aorta was cleaned of adherent tissue and cut into 8 rings, each approx. 2–3 mm long. Special care was taken not to damage the endothelium. The rings were mounted between two hooks in water-jacketed (37 °C) chambers containing PSS bubbled with a mixture of 95% O_2_ and 5% CO_2_ at pH 7.4. The composition of PSS was (in mmol/l): NaCl 118.0, KCl 4.7, KH_2_PO_4_ 1.2, MgSO_4_ 1.2, CaCl_2_ 2.5, NaHCO_3_ 25.0 and glucose 11.0. The preparations were connected to an isometric force transducer (Experimetria Hungary), stretched passively to 20 mN and equilibrated for 60 minutes.

After the equilibration period, SMe1EC2 was applied to the bath in cumulative concentrations of 1×10^–8^–1×10^–4^ mol/l. Then the drug was washed out and the preparations were precontracted with high potassium PSS (KPSS, 100 mmol/l) in which NaCl in normal PSS was exchanged for an equimolar concentration of KCl. When the contraction reached a steady state, SMe1EC2 was added in cumulative manner. In a different set of experiments, contraction was induced by supramaximal concentration of phenylephrine (PE, 1×10^–6^ mol/l). At the plateau of the contraction, the effect of acetylcholine in the cumulative concentrations of 1×10^–8^–1×10^–5^ mol/l was tested in absence or presence of SMe1EC2 in the concentration of 1×10^–6^ or 1×10^–5^mol/l. After washing with PSS and reaching the initial tension value, concentration-response curves of phenylephrine (1×10^–9^–1×10^–5^ mol/l) were performed in absence or presence of SMe1EC2 (1×10^–6^ or 1×10^–5^mol/l). The same protocol was used with concentration-response curves of serotonin (1×10^–8^–1×10^–4^ mol/l). Values of pD_2_ (logarithm of IC_50_ which represents the concentration of a drug required for 50% inhibition *in vitro*) were calculated on using GraphPad Prism.

### Statistical analysis

The results are presented as the mean ± SEM. Statistical analyses were performed by using the unpaired Student's t-test or the two-way ANOVA with Bonferroni posttest. A value of *p<*0.05 was considered statistically significant.

## Results

**Figure 1:**
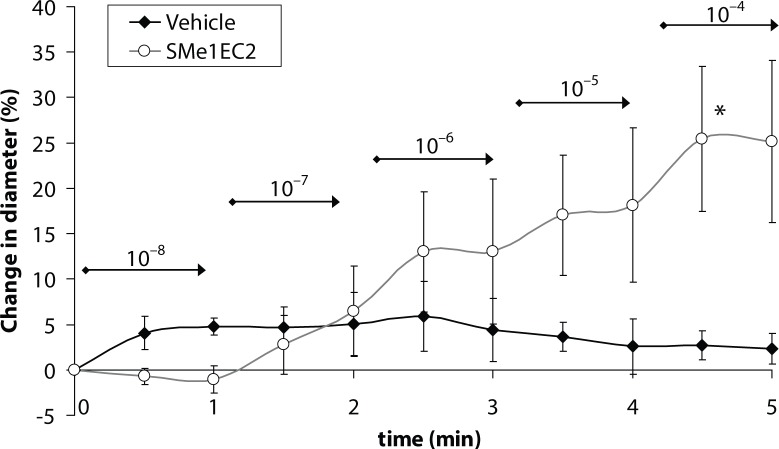
Cerebral artery. Effect of SMe1EC2 applied in rising concentrations of 1×10^–8^ to 1×10^–4^ mol/l (indicated above arrows in mol/l) on the internal diameter of isolated cerebral arterioles. Data are means ± S.E.M., n=3–4, compared with vehicle application (Krebs), *p<0.05.

**Figure 2:**
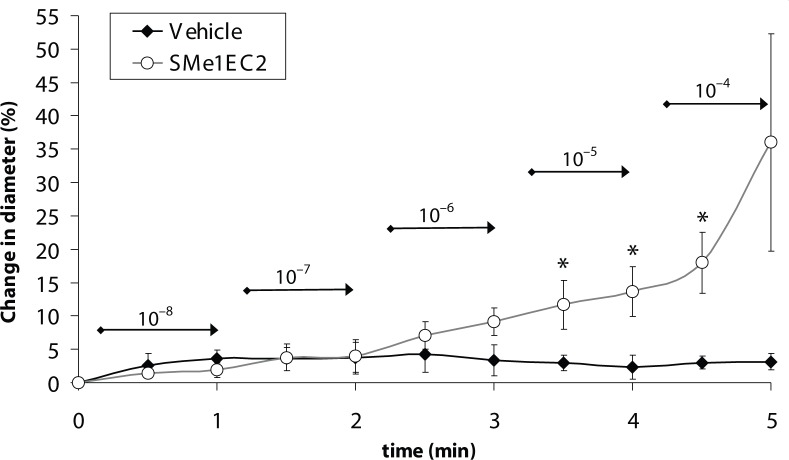
Coronary artery. Effect of SMe1EC2 applied in rising concentrations of 1×10^–8^ to 1×10^–4^ mol/l (indicated above arrows in mol/l) on the internal diameter of isolated coronary arterioles. Data are means ± S.E.M., n=3–4, compared with vehicle application (Krebs), *p<0.05.

Abluminimal application of SMe1EC2 elicited dose-dependent dilatations of isolated cerebral arterioles reaching statistically significant dilatation (25±8%) at the highest concentration (1×10^–4^ mol/l) (Figure 1). In the coronary arterioles, SMe1EC2 induced a significant dilatation in the concentrations of 1×10^–5^ and 1×10^–4^ mol/l (Figure 2). The maximal dilation (18±5%) of the vessel induced by the highest concentration (1×10^–4^ mol/l) of SMe1EC2 was reached after 4.5 minutes.

**Figure 3:**
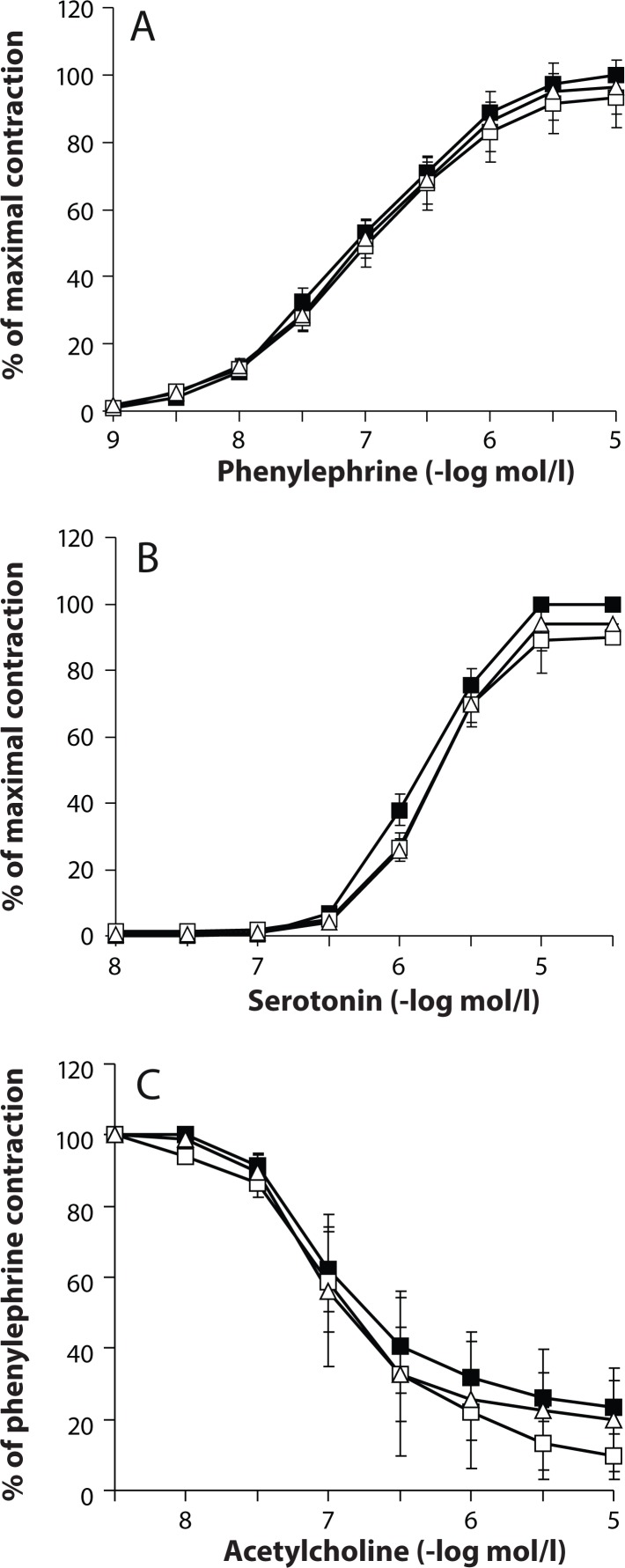
Aorta. Effect of SMe1EC2 on concentration-response curves of phenylephrine (A), serotonin (B) and acetylcholine (C). ■ – control response in the absence of SMe1EC2, □ – response in the presence of SMe1EC2 1×10^–6^ mol/l, Δ – response in the presence of SMe1EC2 1×10^–5^ mol/l. Data are means ± S.E.M., n=7–8.

In the aorta, however, even the high concentrations used failed to affect either the basal tonus or KPSS-induced increased tonus. As shown in Figure 3, SMe1EC2 in the concentrations of 1×10^–6^ or 1×10^–5^mol/l did not influence the concentration-response curves either of phenylephrine or serotonin. The maximal responses to these agents and the pD_2_ values (Table 1) were not affected. Neither did SMe1EC2 change the endothelium-dependent relaxation induced by stimulation of M_3_ muscarinic receptors with acetylcholine.

**Table 1:** pD2 values of the concentration-response curves to phenylephrine and serotonin.

	Phenylephrine	Serotonin
Control	7.509±0.102	6.37±0.117
SMe1EC2 1×10^–6^mol/l	7.527±0.155	6.317±0.202
SMe1EC2 1×10^–5^mol/l	7.518±0.145	6.214±0.135

## Discussion

In our previous experiments, SMe1EC2 improved vascular endothelial function damaged *in vivo* by diabetes or *in vitro* by hyperglycemic PSS (Zurova-Nedelcevova *et al.* 2006). Simulation of hyperglycemia in *in vitro* conditions by incubation of vessels or endothelial cells in physiological solution containing high concentration of glucose (44 mmol/l) leads to increased production of reactive oxygen species (ROS) (Yano *et al.* 2004) presenting as an injury of acetylcholine-induced vessel relaxation. In this study, we raised the possibility that the beneficial effects of SMe1EC2 were attributed to mechanisms intrinsic to the vascular wall.

To this end, we first assessed the direct effect of SMe1EC2 on contractile and relaxant function of the rat aorta. We found that SMe1EC2 in the concentrations of 1×10^–6^ or 1×10^–5^ mol/l had no effect on the basal tone and high potassium-induced aortic contraction. This indicates that the drug tested up to 1×10^–5^mol/l did not interfere with mechanisms of vascular depolarization or calcium entrance. We also studied the possibility that SMe1EC2 affects receptor-mediated aortic relaxation and/or contraction. SMe1EC2, at the concentrations of 1×10^–6^ and 1×10^–5^ mol/l, did not influence the acetylcholine relaxation mediated by muscarinic M_3_ receptors. Moreover, it did not influence the concentration-response curves of phenylephrine or serotonin, suggesting that it possesses no or minimal α_1_-adrenergic or 5-HT-antagonistic activity. On balance, we suggest that SMe1EC2 had no direct vasomotor effect on the rat aorta.

It has been suggested that free-radical scavengers may be responsible for changes in endothelial-dependent vasomotion. Under physiological conditions, vascular endothelium produces nitric oxide and superoxide anion radical (^•^O_2_^–^), which are responsible for maintaining resting tone in arteries. Increase in one of these mediators leads to the changes in arterial tone. Endothelium-dependent relaxation induced by acetylcholine was found to be slightly increased by superoxide dismutase (SOD) (Esenabhalu *et al.* 2002), suggesting that superoxide radical scavengers may improve acetylcholine-induced relaxation. These findings are in agreement with our observation that SMe1EC2, lacking the ability to scavenge superoxide radical, had no influence either on basal tone or on acetylcholine-induced relaxation.

Further we evaluated the effect of SMe1EC2 on resistance arteries. Our results showed a slight but significant vasodilator effect of SMe1EC2 on rat microvessels, yet the effect was observed only at the highest concentrations studied. While the internal diameter of isolated coronary arterioles increased in response to exogenous administration of SMe1EC2 (1×10^–5^ mol/l), the diameter of isolated cerebral arterioles was increased only by the highest concentration used (1×10^–4^ mol/l).

In the contradiction to the aorta, which contains an excess of α_1_-adrenoreceptors (Freitas *et al.*, 2003), adrenergic regulation of coronary vasomotion is balanced between α_1_-adrenergic-mediated constriction (or α_2_-adrenergic activation predominating in arterioles) and β_2_-adrenergic–mediated relaxation, which appears to be partially endothelium-mediated (Barbato *et al.*, 2004). We speculate that the vasodilation response of arterioles to high concentrations of the pyridoindole tested may be explained by its action on β_2_-adrenergic receptors, however the exact mechanism of this effect needs to be elucidated. The effect of the highest concentration (1×10^–4^ mol/l) on the diameter of isolated coronary and cerebral arterioles may be operative by its non-specific membrane-stabilizing action.
